# Human Neutrophils Present Mild Activation by Zika Virus But Reduce the Infection of Susceptible Cells

**DOI:** 10.3389/fimmu.2022.784443

**Published:** 2022-06-07

**Authors:** Juliana Bernardi Aggio, Bárbara Nery Porto, Claudia Nunes Duarte dos Santos, Ana Luiza Pamplona Mosimann, Pryscilla Fanini Wowk

**Affiliations:** ^1^ Laboratório de Virologia Molecular, Instituto Carlos Chagas, Fundação Oswaldo Cruz (FIOCRUZ), Curitiba, Brazil; ^2^ Department of Medical Microbiology and Infectious Diseases, University of Manitoba, Winnipeg, MB, Canada; ^3^ Biology of Breathing Group, Children’s Hospital Research Institute of Manitoba, Winnipeg, MB, Canada

**Keywords:** neutrophils, Zika virus, innate immunity, co-culture, neutrophils extracellular traps (NETs), migration

## Abstract

The emergence of the *Zika virus* (ZIKV) has highlighted the need for a deeper understanding of virus-host interactions in order to pave the way for the development of antiviral therapies. The present work aimed to address the response of neutrophils during ZIKV infection. Neutrophils are important effector cells in innate immunity implicated in the host’s response to neurotropic arboviruses. Our results indicate that human neutrophils were not permissive to Asian or African ZIKV strain replication. In fact, after stimulation with ZIKV, neutrophils were mild primed against the virus as evaluated through CD11b and CD62L modulation, secretion of inflammatory cytokines and granule content, production of reactive oxygen species, and neutrophil extracellular traps formation. Overall, neutrophils did not affect ZIKV infectivity. Moreover, *in vitro* ZIKV infection of primary innate immune cells did not trigger neutrophil migration. However, neutrophils co-cultured with ZIKV susceptible cell lineages resulted in lower cell infection frequencies, possibly due to cell-to-cell contact. *In vivo*, neutrophil depletion in immunocompetent mice did not affect ZIKV spreading to the draining lymph nodes. The data suggest that human neutrophils do not play an antiviral role against ZIKV *per se*, but these cells might participate in an infected environment shaping the ZIKV infection in other target cells.

## Introduction

*Zika virus* (ZIKV) is an enveloped vector-borne RNA virus, a member of the genus *Flavivirus* that includes important human pathogens. The epidemic potential of flaviviruses is linked to the global distribution of their arthropod vectors (mainly *Aedes* spp.), as well as human population density, mobility, and anthropogenic interventions (1). Furthermore, the mutation rate in the viral genome and the host immune status may also impact viral spread and pathogenesis (2). Since 2007, after decades of sparse reports of infection in Africa and Asia, the Asian genotype of ZIKV has been implicated in outbreaks in human populations; from Southeast Asia, it has spread throughout the Americas and has reached Europe (3). In Brazil, ZIKV was first detected in 2015 and categorized as a Public Health Emergency in 2016 (4). More than 220,000 cases were notified, and the infection has been associated with congenital diseases and Guillain-Barré Syndrome in adults (5–7).

ZIKV infection has been reported to trigger rapid recruitment and activation of monocytes, NK cells, plasmacytoid dendritic cells, and lymphocytes, and the upregulation of multiple signaling pathways, such as pro-inflammatory cytokines and chemokines in the blood of non-human primates and humans (8–13). Among the innate immune cells, monocytes, dendritic cells, and macrophages have been described as targets of ZIKV infection and replication (14–17). It has been suggested that the ensuing innate immune response can be associated with the fate of the ZIKV disease. Cell infiltration and inflammation at ZIKV infection sites contributed to placental dysfunctions (18) and encephalitis (19–21). ZIKV affects the adhesive properties of monocytes, enhancing their transmigration through endothelial barriers and viral dissemination to neural cells (22). Moreover, neutrophils, Ly6C^mid-hi^ monocytes, and CD45^+^ monocytes from AG129 mice (type I and II IFN receptor-deficient), and bone marrow-derived S100A4+ macrophages from AG6 mice (type I, II, and III IFN receptor-deficient) were shown to be essential for ZIKV dissemination and pathogenesis in peripheral organs and testis (23–25). CD45^+^CD11b^+^ monocytes and macrophages play an important role in inhibiting ZIKV spread in the placenta (26), while infected human placental macrophages might gain access to the fetus (27). In this respect, the role played by neutrophils during ZIKV infection remains undetermined. Elucidating mechanisms by which neutrophils mediate an antiviral response may enable the development of therapies that retain antiviral functions but limit inflammation-associated damage.

Mature neutrophils are the most abundant granulocytes in the human bloodstream and the main effectors during inflammation and infection. Once in the infection site, neutrophils can rapidly eliminate intra- and extracellular pathogens by phagocytosis, oxidative burst, multiple granule proteolytic enzymes, antimicrobial peptides, and neutrophil extracellular traps (NETs) release (28, 29). Neutrophils have also been recognized as multitasking cells capable of cross-talking with adaptive responses, for example, presenting antigens during viral infections (30, 31). The relevance of neutrophils during flavivirus infections was demonstrated for *West Nile virus* (WNV) infections, where neutrophils act as Trojan horses carrying the virus into the central nervous system (CNS), enhancing the WNV neuroinvasive disease (32). Neutrophil depletion prior to WNV infection resulted in reduced viremia and enhanced host survival (33).

Here, we address the role of neutrophils on ZIKV pathogenesis by means of the *in vitro* screening of classical human neutrophil defense mechanisms after stimulation with different ZIKV strains. We report that neutrophils are not targets for ZIKV replication, nor are they a good responder to ZIKV, yet they reduce ZIKV infection when in contact with target cells.

## Materials and Methods

### Cells

**Figure 1:**
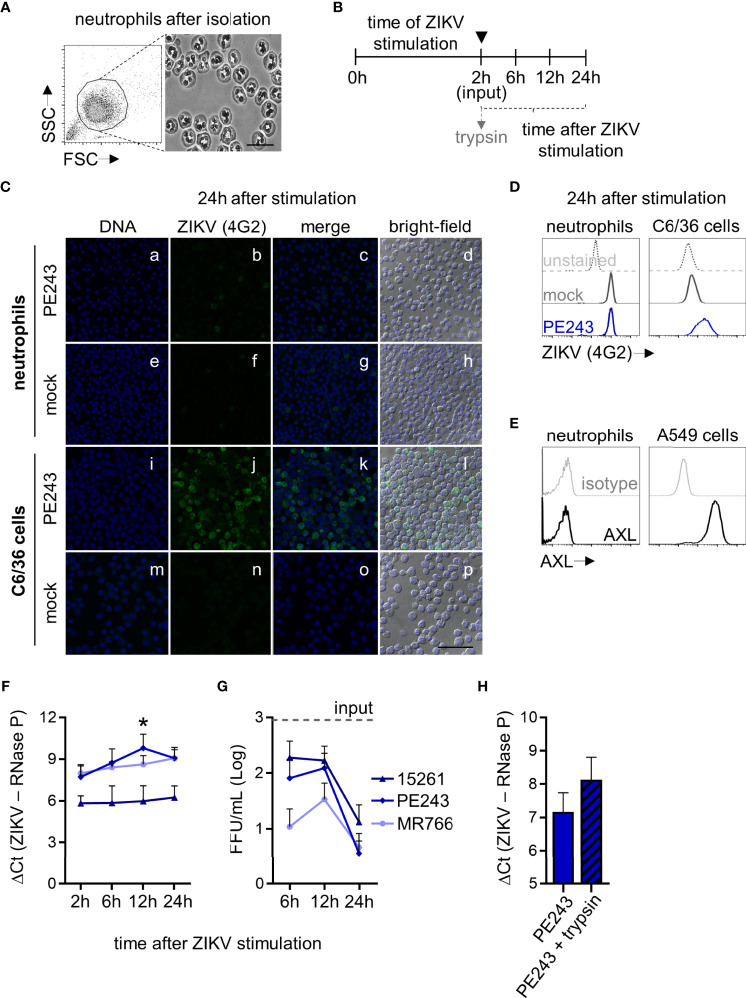
ZIKV does not replicate in human neutrophils. **(A)** Neutrophils morphological features after isolation shown by flow cytometry and microscopy (bright-field, bar = 25 µm, magnification = 100x.). **(B)** The cells were stimulated *in vitro* for 2 hours with ZIKV BR 2015/15261, ZIKV PE243, ZIKV MR766 (1 MOI), or mock (C6/36 cells-conditioned media in an equivalent volume of ZIKV strains). Then, neutrophils were washed to remove the stimuli (indicated by ▼) and evaluated directly after (2 hours) and at 6, 12, and 24 hours after stimulation. When indicated, neutrophils were treated with trypsin after the wash step at 2 hours of stimulation with ZIKV strains. **(C)** Immunostaining of neutrophils with 4G2 (E protein; green) at 24 hours after ZIKV PE243 (a-d) or mock (e-h) stimulation. Nuclei (blue) were stained with Vybrant DyeCycle Violet. Merge of these stains and the bright-field colocalization are also shown. C6/36 cells (l-p) were used as a positive infection control (bar = 50 µm, magnification = 40x.) **(D)** Histograms showing 4G2 intensity of fluorescence in neutrophils and C6/36 cells at 24 hours after mock and ZIKV PE243 stimulation, as measured through flow cytometry. Infected-unstained cells were used as a negative fluorescence control. **(E)** Histograms showing AXL receptor intensity of fluorescence in neutrophils and A549 cells. An isotype control antibody was used as a negative fluorescence control. One result that is representative of three independent experiments is shown. **(F)** RNA levels of ZIKV strains in neutrophils at 2, 6, 12, and 24 hours after stimulation. **(G)** ZIKV loads on neutrophil culture supernatant at the same time after stimulation with ZIKV strains. The dashed line named input represents the mean of the titers of the three ZIKV strains that were still detected on the supernatant after stimuli removal after 2 hours of stimulation. **(H)** RNA levels of ZIKV PE243 detected in neutrophils treated or not with trypsin after 2 hours of stimulation. Bars indicate the standard error of the mean (SEM). Three-four independent experiments are shown (n = 9-15). The asterisk (*) denotes the statistical difference between 6 and 12 hours of the ZIKV PE243 stimulation.

Human peripheral blood was obtained *via* an intravenous puncture from healthy volunteers (both genders, aged between 21 and 50 years old and with no clinical evidence of disease) upon their written consent. The procedures were in compliance with the determinations of the Conselho Nacional de Ética em Pesquisa-CONEP [National Research Ethics Committee] (CAAE 60643816.6.0000.5248). Human neutrophils were isolated from peripheral blood by negative selection with magnetic microspheres, MACSxpress Neutrophil Isolation Kit, and MACSxpress Separator (Miltenyi Biotec), all according to the manufacturer’s instructions. Cell viability was determined by the Trypan blue exclusion assay, and neutrophil purity was confirmed by cytospin slides (Cytospin 4; Thermo Fisher Scientific) visualized through microscopy (LEICA AF6000 Modular System) (**Figure 1A**) and flow cytometry (FACS Canto II - BD Biosciences).

Peripheral blood mononuclear cells (PBMCs) were isolated using Histopaque at a density of 1.077 g/mL (Sigma-Aldrich). CD14^+^ cells were sorted with the MACS system (CD14 human MicroBeads, Miltenyi Biotec), according to the manufacturer’s instructions, and were seeded at 5x10^5^ cells/mL, 96-well plate, in RPMI-1640 media with L-glutamine (Lonza) supplemented with 10% of fetal bovine serum (FBS; Gibco), 25 µg/mL gentamicin (Gibco), 100 IU/µg/mL penicillin-streptomycin (Sigma-Aldrich), 12.5 ng/mL recombinant human GM-CSF (PeproTech), and 25 ng/mL recombinant human IL-4 (PeproTech). The cells were incubated for 7 days at 37°C, with 5% CO_2_ and a humid atmosphere. On the third day of incubation, a fresh supplemented medium was added to the cell culture. Differentiation of human monocyte-derived dendritic cells (mdDCs) was confirmed by flow cytometry (CD11c^+/high^CD14^+/low^).

*Aedes albopictus* mosquito C6/36 cells (ATCC CLR-1660) were grown in Leibovitz’s media (L-15; Gibco) supplemented with 5% FBS, 25 µg/mL gentamicin, and 0.26% tryptose (Sigma-Aldrich) at 28°C. Human A549 lung epithelial cell line (ATCC CCL-185), JEG-3 placental cell line (ATCC HTB-36), and SH-SY5Y neuroblastoma cell line (ATCC CRL-2266) were maintained in RPMI-1640 media supplemented with 10% FBS, 25 µg/mL gentamicin, and 100 IU/µg/mL penicillin-streptomycin at 37°C, 5% CO_2_, and humid atmosphere.

### Zika Virus

Viral stocks of the ZIKV Asian strains, the clinical isolates BR 2015/15261 (34) and PE243 (35), and the ancestral ZIKV African isolate MR766 (36) were prepared in C6/36 cells. Seven days after infection, cell culture supernatant was collected, clarified by centrifugation, and later it was titrated by foci-forming immunodetection assay in C6/36 cells (37). In parallel, C6/36 cells were maintained in the same conditions without viral addition; this conditioned supernatant, hereby called mock, was used as a negative control for cell activation and infection.

### Cell Interaction With ZIKV

Neutrophils at 2.5x10^5^ cells/200 µL of RPMI-1640 media supplemented with 25 µg/mL gentamicin, and 100 IU/µg/mL penicillin-streptomycin, 96-well plate, were incubated for 2 hours with ZIKV strains (BR 2015/15261, PE243 and MR766) using a multiplicity of infection (MOI) of 1 at 37°C, 5% CO_2_, and humid atmosphere under agitation. As a control for cell activation, neutrophils were also incubated in the same conditions with mock, 100 ng/mL of *Escherichia coli* lipopolysaccharide (LPS-EK, *In vivo*Gen), or 16 nM of phorbol 12-myristate 13-acetate (PMA; Sigma-Aldrich). After the 2-hour incubation period, neutrophils were washed twice (250 x *g*; 10 minutes) in a non-supplemented media and seeded in 96-well plates in RPMI-1640 media supplemented with 10% FBS and antibiotics at 37°C, 5% CO_2,_ and humid atmosphere. Supernatant collected right after the wash step was denominated input and used as a control to account for any remaining viruses in the neutrophil culture after the initial inoculum had been washed out (**Figures 1B, G**, dashed line). Two (input), 6, 12, and 24 hours after the beginning of the stimulation, neutrophils and the culture supernatant were harvested and analyzed. Supernatants were stored at -80°C until further analysis. In some experiments, in order to exclude non-internalized virus binding on their surface, neutrophils were treated right after the steps involving washing (2 hours/input) with 0.05% trypsin-EDTA (Gibco) (**Figure 1B**) for 10 minutes at room temperature, followed by FBS addition, washed, and suspended in fresh media (38). Additionally, neutrophils were incubated for 6 hours with ZIKV strains (BR 2015/15261, PE243 and MR766), 1 MOI at 37°C, 5% CO_2_, and humid atmosphere under agitation. Supernatant was recovered and incubated with A549, 1x10^5^ cells/well seeded in 24-well plates. After the 2-hour incubation period, cells were washed twice (250 x *g*; 10 minutes) in a non-supplemented media and cells were maintained in RPMI-1640 media supplemented with 10% FBS and antibiotics at 37°C, 5% CO_2,_ and humid atmosphere for 34 hours (**Figure 4** upper panel). Supernatant was collected and virus titers were determined as above.

Chemokines in PBMCs and mdDCs culture supernatants were quantified 24 and 48 hours after ZIKV stimulation. For that, PBMCs (after isolation) and mdDCs (after 24 hours of resting) at 1x10^6^ cells/500 µL were stimulated with the same protocol described above for neutrophils (**Figure 1B**) but were seeded in 24-well plates.

A549, C6/36, JEG-3, and SH-SY5Y cell lines, 1x10^5^ cells/well seeded in 24-well plates, were incubated for 2 hours with ZIKV strains (1 or 10 MOI) (or different stimuli where otherwise indicated) in 400 µL of media without FBS. After the incubation period, cells were washed twice with non-supplemented media and kept in a media supplemented with 10% FBS and antibiotics during the indicated times.

### Flow Cytometry

The viability of the neutrophils and A549 cells was determined at the indicated time points using Annexin V (31490016; ImmunoTools) and 7-Aminoactinomycin D (559763; 7-AAD; BD Bioscience) following the manufacturers’ instructions (Annexin V Apoptosis Detection Kit; BD Bioscience). The frequency of ZIKV antigen in neutrophils, A549, C6/36, JEG-3, and SH-SY5Y cell lines was measured by staining with a flavivirus group-specific envelope protein (E), monoclonal antibody 4G2 (ATCC HB-112) (37). Briefly, the cells were recovered and, if necessary, they were detached from the flask containing the cell culture (A549 cells with trypsin and C6/36 with cell scraper) and blocked (5% FBS and 1% human AB serum in PBS) for 20 minutes at room temperature. Next, cells were fixed and permeabilized using the Cytofix/Cytoperm Fixation/Permeabilization Kit (554714; BD Biosciences), stained with a 4G2 FITC-conjugated antibody for 45 minutes at 37°C, and washed twice. Alternatively, these cells were labeled with 4G2 antibody, goat anti-mouse Alexa Fluor 488 secondary antibody (A-10680; Thermo Fisher Scientific), and Vybrant DyeCycle Violet Stain (V35003; Thermo Fisher Scientific) and fixed in slides that had previously been treated with poly-L-lysine (Sigma-Aldrich) for confocal microscopy imaging (LEICA SP5 AOBS). For surface marker staining, neutrophils were blocked and incubated with fluorochrome-conjugated mouse anti-human monoclonal antibodies specific for CD11b (557754; clone ICRF44), CD16 (557743; clone 3G8), CD62L (561915; clone DREG-56) (BD Biosciences), and Hu Axl (12-1087-42; clone DS7HAXL) (eBioscience) for 20 minutes at room temperature. To measure the generation of intracellular reactive oxygen species (ROS), neutrophils were labeled with 0.5 µM of 5-(and-6)-chloromethyl-2’,7’-dichlorodihydrofluorescein diacetate, acetyl ester (CM-H_2_DCFDA) probe (C6827; Invitrogen) for 45 minutes at 37°C. Cytokines and chemokines in the supernatant of neutrophils, PBMCs, and mdDCs culture supernatants were quantified using the Human Inflammatory Cytokine Cytometric Bead Array I Kit (551811; BD Biosciences) and Human Chemokine Cytometric Bead Array Kit (552990; BD Biosciences), following the manufacturer’s instructions. Flow cytometry was performed on a FACS Canto II with BD FACSDiva software (BD Biosciences), and the resulting data were analyzed on FlowJoV10 (BD Biosciences).

### RT-qPCR

Neutrophil RNA was extracted using the RNeasy Mini Kit (74104; Qiagen) according to the manufacturer’s instructions. RT-qPCR was performed to detect ZIKV in a 20 μL final volume of reaction containing GoTaq one-step RT-qPCR master mix (Promega), 25 ng of sample RNA, 500 nM of the ZIKV1086 and ZIKV1162c oligonucleotides, and 200 nM of ZIKV1107-FAM probe, according to a previously described protocol (39). RNase P (*RPPH1*) was used as a reference gene (40). All reactions were carried out in a LightCycler 96 System, and the fluorescence threshold limit of the probe was automatically set by LightCycler software (Roche). The results were represented as the ΔCt between ZIKV and RNAase P amplification.

### Neutrophil Elastase Measurement

Elastase was measured using the Human PMN-Elastase ELISA Kit (BMS269; Invitrogen) in a neutrophil culture supernatant 6 hours after stimulation. Cell culture supernatant (100 µL) was added to a microwell plate coated with an anti-human polymorphonuclear (PMN) elastase polyclonal antibody and incubated at room temperature for 1 hour with a Horseradish peroxidase-conjugated anti-α 1-proteinase inhibitor antibody. The immune complex was detected by adding tetramethyl-benzidine substrate solution, and the absorbance was determined at 450 nm on a microplate reader (BioTek Synergy H1 Hybrid).

### Neutrophil Extracellular Traps Assessment

Neutrophils at 2x10^5^ cells/200 µL of RPMI-1640 media supplemented with antibiotics were incubated for 5 hours with either mock, ZIKV strains (1 MOI), or 160 nM of PMA in poly-L-Lysine coated Lab-Tek chamber slides (Thermo Fisher Scientific) at 37°C, 5% CO_2_ and a humid atmosphere. For NETs visualization, the supernatant was carefully removed, and the cells were fixed with 3% paraformaldehyde (Sigma-Aldrich) and stained with a rabbit anti-acetyl-histone H3 polyclonal antibody (06-599; Sigma-Aldrich) followed by a goat anti-rabbit Alexa Fluor 488 secondary antibody (710369; Thermo Fisher Scientific) and a Vybrant DyeCycle Violet Stain for 45 minutes at 37°C. The slides were sealed with n-propyl gallate (Sigma-Aldrich) and observed through confocal microscopy (LEICA SP5 AOBS). For NETs quantification, following the stimulation period, neutrophils were treated with 0.04 U/µL of Turbo DNAse (Thermo Fisher Scientific) for 10 minutes at 37°C. The enzymatic digestion was stopped with 5 mM EDTA (41). The culture was centrifuged (300 x g for 1 minute), and the supernatant was collected and diluted 5-fold. Free double-stranded DNA (dsDNA) was quantified using a Quant-IT PicoGreen dsDNA kit (P11496; Invitrogen) in a Qubit 2.0 fluorometer (Invitrogen) according to the manufacturer’s recommendations. To evaluate the effect of pre-formed PMA-induced NETs on viral particle capture, neutrophils were stimulated for 5 hours with media or PMA in the same conditions described above. Then, the culture was stimulated for 1 hour with ZIKV strains (1 MOI). Next, neutrophils were centrifuged (300 x g for 1 minute), and the supernatant was collected and quantified by foci-forming immunodetection assay in C6/36 cells.

### Neutrophil Chemotaxis Assay

Neutrophils at 3x10^5^ cells/200 µL of RPMI-1640 media supplemented with antibiotics were seeded on a 3 µm pore Thin Cert insert (Greiner Bio-One) coupled to a 24-well plate. Inducers of neutrophil chemotaxis were added to the bottom well, 1,000 or 50,000 pg of recombinant human (rh-) IL-8 (PeproTech) in 600 µL of RPMI-1640 media or A549 cell culture previously infected for 48 hours with mock or ZIKV PE243 (1 MOI). For this specific experiment, infected A549 cells were maintained in the absence of FBS. RPMI-1640 media and A549 cells stimulated with mock were used as a negative control for cell migration. After 2 hours at 37°C, 5% CO_2,_ and a humid atmosphere, migratory neutrophils were collected from the bottom well system, and the cell concentration was determined by Turk dye counting. The chemotactic index was calculated as the ratio of the number of migratory neutrophils in each condition divided by the number of neutrophils that migrated in the negative control (42).

### Co-Culture Assay

A549, JEG-3, and SH-SY5Y cells (seeded in 24-well plates) at 1x10^5^ cells/400 µL in RPMI-1640 media supplemented with antibiotics were stimulated for 2 hours with mock or ZIKV strains (1 or 10 MOI) in the absence or presence of neutrophils at a ratio of 1:5. Afterward, neutrophils were assessed for surface markers by flow cytometry as described above, and cells were washed twice and kept in 500 µL of RPMI-1640 media supplemented with 10% FBS and antibiotics for 16 hours. At the end of the 18 hours of infection, cells were assessed for viability or intracellular ZIKV antigen by flow cytometry. To evaluate if neutrophils were physically interacting with A549, JEG-3, and SH-SY5Y cells through surface proteins in some experiments neutrophils were pretreated with trypsin for 10 minutes at room temperature, FBS was added, and then they were washed and suspended in a fresh media and added to the cells in culture. In a different co-culture experiment, A549 cells were incubated for 2 hours with mock or ZIKV strains (1 MOI), washed twice, and incubated for 24 hours. Then, neutrophils at a ratio of 1:5 were either added to or not to these cultures for 16 hours. At the end of the 40 hours of infection, A549 cells were evaluated for the frequency of the ZIKV antigen.

### *In Vivo* ZIKV Infection Model

C57BL/6 mice were obtained from Instituto Carlos Chagas/FIOCRUZ– PR animal facility, and maintained and handled according to the directives of the Guide for the Care and Use of Laboratory Animals of the Brazilian National Council of Animal Experimentation. The protocols were approved by the Committee on the Ethics of Animal Experimentation from Fundação Oswaldo Cruz – CEUA/FIOCRUZ (license LW 03-19). Both male and female mice, 8–12-week-old, were infected subcutaneously in the hind footpad with ZIKV PE243 (5x10^5^ FFU, 10 µL) to determine systemic ZIKV titers. After 10 minutes, 1, 3, 6, and 24 hours of infection, spleen, kidney, and lymph nodes (popliteal (pLN), lumbar aortic (laLN), and sciatic lymph nodes (sLN)) were aseptically removed, homogenized using a tissue grinder, submitted to three freeze-thaw cycles, and the viral load was then measured by foci-forming immunodetection assay in C6/36 cells. Each animal’s ipsi- and contralateral lymph nodes were pooled together. Blood was collected through a cardiac puncture at the same time points, and plasma viremia was titrated in C6/36 cells.

To evaluate the neutrophil influence in ZIKV spread to peripheral organs, neutrophils were depleted, and the animals were infected with ZIKV. Animals were inoculated intraperitoneally with 200 µL of PBS containing 400 µg of anti-mouse Ly6G (BE0075-1; clone 1A8; BioxCell) or mouse IgG2a isotype control (BE0085; clone C1.18.4; BioxCell). Control animals received 200 µL of PBS (Lonza). After 18 hours, the frequency of PMNs in total blood was evaluated through flow cytometry by surface staining with fluorochrome-conjugated anti-mouse monoclonal antibodies specific for CD11b (552850; clone M1/70) and Ly6C/G (553129; clone RB6-8C5) (BD Biosciences). Neutrophil-depleted animals were inoculated in the hind footpad with PBS only or 1,000 ng of LPS (to induce an inflammatory environment). After 3 hours, mice were inoculated with 10 µL of ZIKV PE243 (1.3x10^6^ FFU) in the same footpad. One hour later, popliteal lymph nodes (pLNs) were pooled, harvested, and the viral titer was determined.

### Statistical Analyses

Analyses were performed using GraphPad Prism 8 (GraphPad Software, Inc.). Wilcoxon matched-pairs signed-rank test (nonparametric paired t-test) was used in the analysis of the *in vitro* experiments with primary human cells to clarify individual patterns. One-way ANOVA with Tukey’s multiple comparison test was used in animal experiments to compare the average of groups. A cut-off of p <0.05 was considered significant.

## Results

### ZIKV Does Not Establish a Productive Infection in Human Neutrophils

We first evaluate whether any of the tested ZIKV strains trigger human neutrophil death until 24 hours after stimulation (**Supplementary Figure 1**), following the experimental setting in **Figure 1B**. A progressively higher exposition of phosphatidylserine was observed over time for all samples with some small differences between mock- and the ZIKV strains-stimulated neutrophils, however low loss of cellular integrity was indicated by 7-AAD uptake (**Supplementary Figure 1**). The observed neutrophil phenotype concurs with previous knowledge that, in the absence of inflammation, neutrophils have a short life span and undergo constitutive apoptosis (43) and LPS, which is a positive control of neutrophil activation, restricts apoptosis at the late time points (44).

In addition to subverting the target cell into a reservoir for replication and dissemination, viruses may use the infected cells as a Trojan horse to overcome physiological host defense barriers (32). The infection can also result in the inhibition of important cell signal transduction pathways (17). To address this issue, we sought to understand whether human neutrophils are susceptible to ZIKV infections and whether they can sustain them. No positive cells for the intracellular staining of the ZIKV E protein (4G2 antibody) were detected 24 hours after stimulation with the three ZIKV strains tested through immunofluorescence or flow cytometry (**Figures 1C, D**). Such findings stand in contrast with the infection observed in the highly permissive mosquito cell line C6/36 (**Figures 1C, D**). The possibility of an active internalization of a few viral particles by neutrophils *via* receptors or phagocytosis could not be neglected, even though we reported the absence of AXL on the surface of human neutrophils, which was expressed in the ZIKV susceptible human lineage A549 (**Figure 1E**). Indeed, ZIKV RNA could be detected in neutrophils incubated with the viruses (**Figure 1F**). However we did not observe a significant increase in ZIKV RNA levels over time (**Figure 1F**), nor did we observe the release of functional viral particles in loads greater than what was found in the input (**Figure 1G**). Treatment of neutrophils with trypsin after the wash steps reduced but did not expunge virus RNA levels (**Figure 1H**). This suggests that the low RNA levels measured cannot be entirely attributed to viral inoculum remnants, and could be explained, if only partially, by the virus particles that adhere to the cell’s membranes.

### Human Neutrophils Are Mildly Responsive to Direct Contact With ZIKV

**Figure 2:**
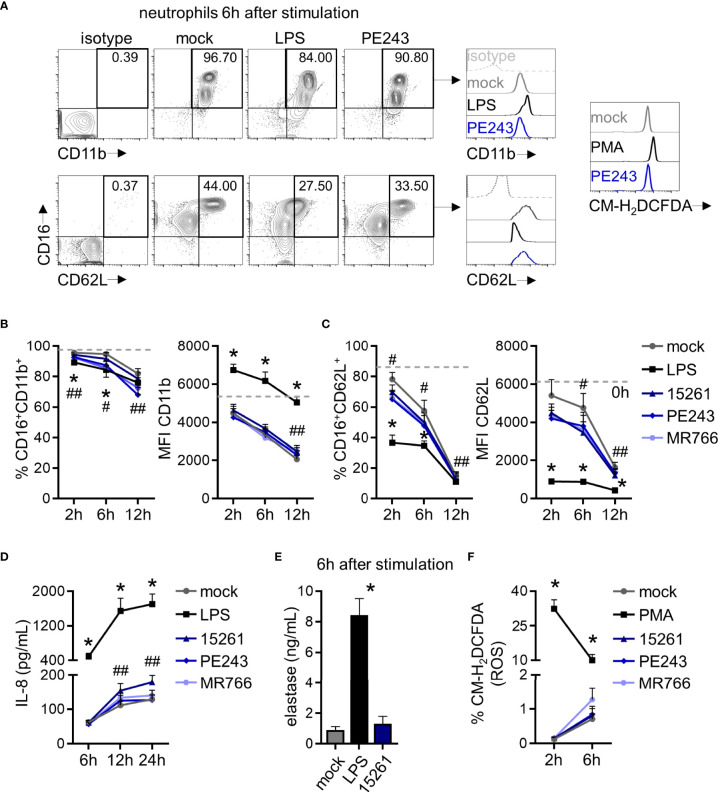
ZIKV mildly modulates CD62L expression in human neutrophils but does not stimulate the production of cytokines, elastase, and reactive oxygen species by these cells. **(A)** Contour plots depicting the frequency of neutrophils CD16^+^CD11b^+^ and CD16^+^CD62L^+^ and the fluorescence intensity of CD11b and CD62L molecules inside these populations at 6 hours after stimulation with mock, LPS (100 ng/mL), or ZIKV PE243 (1 MOI). This time point and strain were selected as a representative of these results. Isotype control antibodies were employed as a negative fluorescence control to set the gates. A representative result of chloromethyl-H_2_DCFDA (CM- H_2_DCFDA) fluorescence in the total neutrophil population at 6 hours after stimulation with mock, PMA (16 nM), or ZIKV PE243 is also shown. **(B)** Frequency of neutrophils CD16^+^CD11b^+^ and the mean fluorescence intensity (MFI) of CD11b in that population at 2, 6, and 12 hours after stimulation with mock, LPS, or ZIKV strains (1 MOI). The dashed line represents the measurements right after neutrophil isolation from blood (time 0). **(C)** Same analysis as in **(B)** was applied to the CD62L molecule. **(D)** IL-8 levels in neutrophil culture supernatant at 6 and 12 hours after stimulation. **(E)** Elastase levels in neutrophil culture supernatant at 6 hours after stimulation. **(F)** Frequency of neutrophils CM-H_2_DCFDA^+^ at 2 and 6 hours after stimulation. Bars indicate SEM. Two-three independent experiments are shown (n = 6–12). The asterisk (*) denotes the statistical difference between mock and LPS or PMA, and the number sign (#) represents the difference between mock and all the three ZIKV strains at that time point. Double number signs (##) denote the statistical difference between mock, ZIKV PE243, and ZIKV MR766 (B - %CD16^+^CD11b^+^); between mock, ZIKV 15261, and ZIKV MR766 (B - MFI CD11b); between mock and PE243 (C - %CD16^+^CD62L^+^); between mock, ZIKV 15261, and ZIKV PE243 (C - MFI CD62L); between mock and ZIKV 15261 (D).

**Figure 3:**
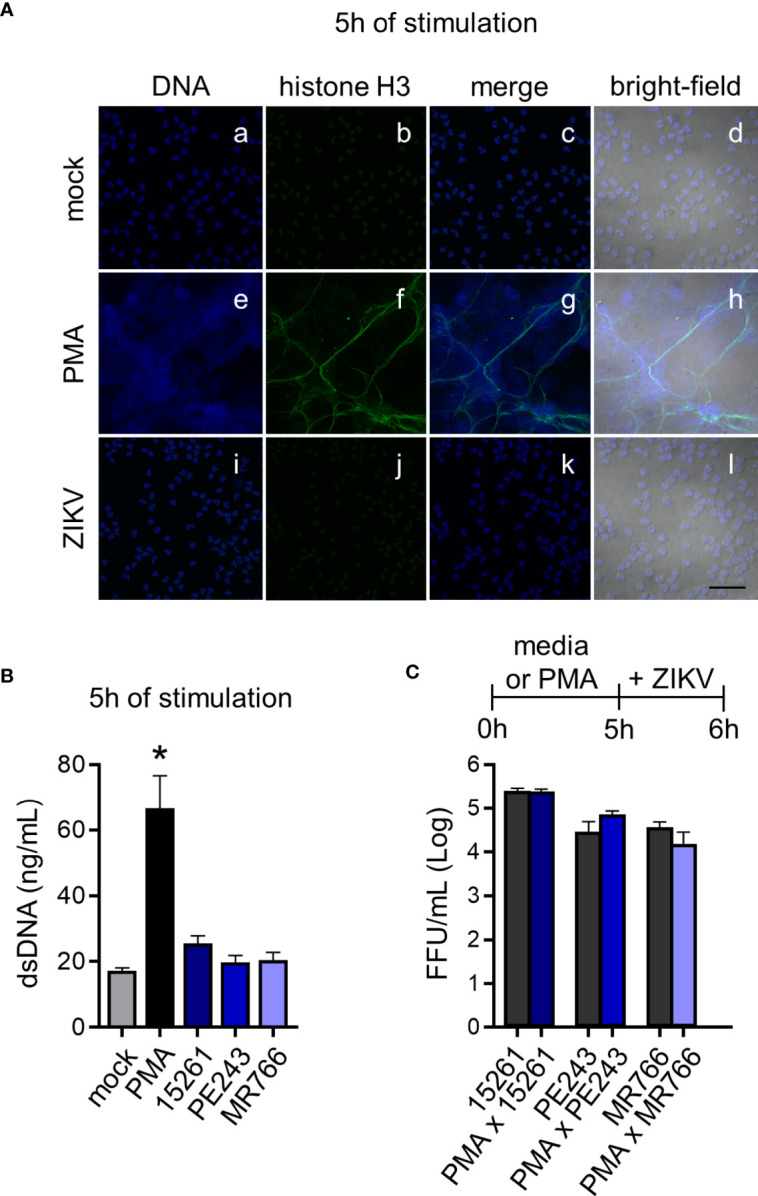
ZIKV does not induce NETs and is not captured by the DNA trap. **(A)** Immunostaining of neutrophils DNA (Vybrant DyeCycle Violet, blue) and acetyl-histone H3 (green) after 5 hours of stimulation with mock (a-d), PMA (160 nM) (e-h), or ZIKV PE243 (1 MOI) (i-l). ZIKV PE243 was chosen as the representative of the ZIKV strains’ effects. Merge of these stains and the bright-field colocalization are also shown (bar = 50 µm, magnification = 60x). One representative of three independent experiments is shown. **(B)** Free double-stranded DNA (dsDNA) in neutrophil culture supernatant after 5 hours of stimulation with mock, PMA, or ZIKV strains (1 MOI). **(C)** ZIKV strain loads on neutrophil culture supernatant after 1 hour in the presence or absence of NETs induced by PMA stimulation during 5 hours. Bars indicate SEM. Two-three independent experiments are shown (n = 6–9). The asterisk (*) denotes the statistical difference between mock and PMA.

**Figure 4:**
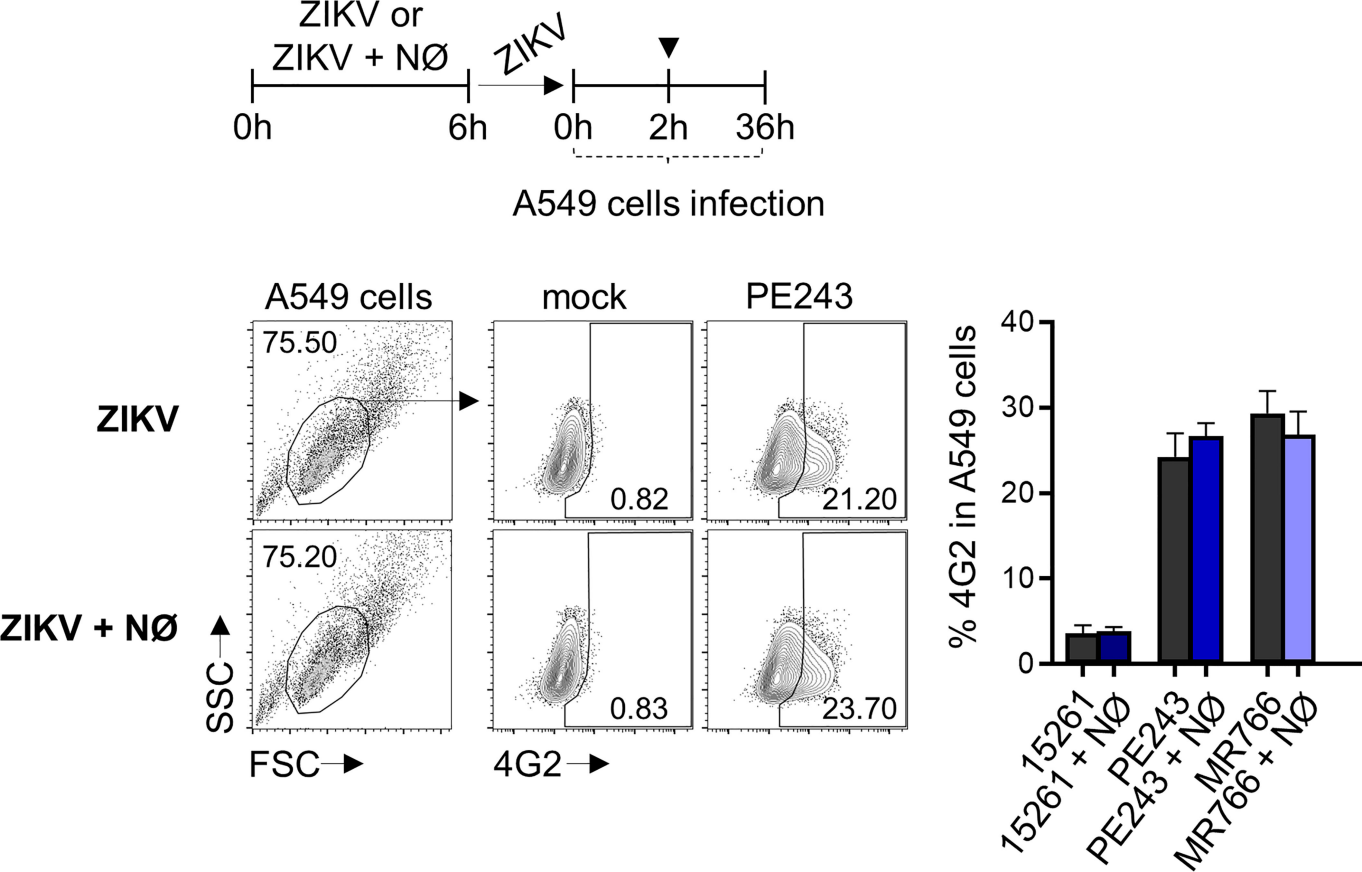
Human neutrophil does not impair ZIKV infectivity. Frequency of 4G2^+^ A549 cells at 36 hours after infection with ZIKV strains (1 MOI) previously incubated for 6 hours in the absence (ZIKV) or presence (ZIKV + NØ) of neutrophils. ZIKV PE243 is shown as a representative of the ZIKV strains’ effects on the flow cytometry plots. The mock condition was used as a negative fluorescence control. Bars indicate SEM. Three independent experiments are shown (n = 9). (▼) indicates the moment A549 cells were washed to remove the virus. NØ, neutrophils.

The recognition of viral elements in the cell cytosol triggers defense mechanisms involved in viral replication, control, and inflammation. However, a significant part of neutrophil activation mechanisms is coordinated through the signaling of cell surface receptors (43). Following the experimental setting in **Figure 1B**, the expression of the adhesion integrin CD11b was upregulated over time, following LPS stimulation, while the selectin adhesion receptor CD62L was downregulated (**Figures 2A-C**), indicating the priming of neutrophils (45, 46). Nevertheless, limited differences were observed in the expression of CD11b and CD62L molecules between mock- and some neutrophils that had been stimulated with ZIKV strains (**Figures 2B, C**). Likewise, significant differences were observed between mock and ZIKV 15261 in the IL-8/CXCL8 levels that neutrophils had secreted after 24 hours of stimulation (**Figure 2D**). Similar results were obtained for IL-1β and IL-6 (data not shown). The low levels of elastase detected in the neutrophils culture supernatant after 6 hours of stimulation with ZIKV BR 2015/15261 were similar to mock-stimulated cells (**Figure 2E**). Overall low production of ROS in neutrophils was also noted after ZIKV stimulation; this was measured by the oxidation of chloromethyl-H_2_DCFDA (**Figures 2A, F**), which contrasts with the oxidative stress generated by PMA stimulation, a well-known ROS inducer. Upon 5 hours of stimulation, we tested and confirmed that NETs were not induced by any ZIKV strains; this was assessed through the absence of web-link extracellular structures colocalizing with DNA and histone (**Figure 3A**) and the absence of free DNA in neutrophil supernatants (**Figure 3B**). PMA is a robust NET inducer over a 3-4-hour course *via* ROS (47), as observed in **Figures 3A, B**. Moreover, ZIKV was not trapped by NETs, as the same loads of ZIKV were quantified in the supernatant of neutrophils that had or had not been stimulated with PMA (**Figure 3C**). To confirm that neutrophils do not impact ZIKV particles, neutrophils were stimulated for 6 hours with ZIKV, and the free virus recovered from the supernatant did not have impaired infectivity in a subsequent infection of susceptible cells (**Figure 4**).

### ZIKV Infection Does Not Provide a Favorable Environment for Human Neutrophil Migration

**Figure 5:**
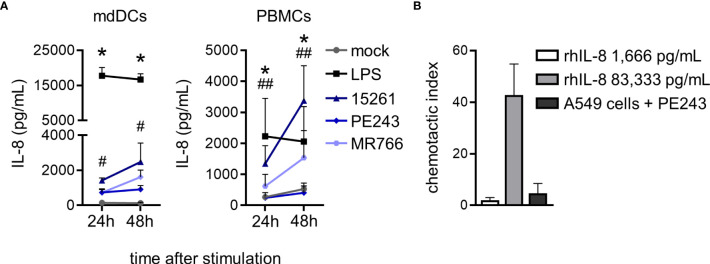
ZIKV infection in primary human cells does not induce high levels of IL-8 to promote migration of human neutrophils. **(A)** IL-8 levels in mdDCs and PBMCs culture supernatant at 24 and 48 hours after stimulation with mock, LPS (100 ng/mL), or ZIKV strains (1 MOI). **(B)** Chemotactic index of neutrophils after 2 hours of stimulation with 1,666 or 83,333 pg/mL of rhIL-8 or the supernatant of A549 cell culture pre-infected with ZIKV PE243 for 48 hours. Bars indicate SEM. Two-three independent experiments are shown (n = 6–9). The asterisk (*) denotes the statistical difference between mock and LPS, and the number sign (#) represents the difference between mock and all the three ZIKV strains at the time point. Double number signs (##) denote the statistical difference between mock and ZIKV 15261 (PBMCs – 24h) and between mock, ZIKV 15261, and MR766 (PBMCs – 48h).

Although neutrophils are circulatory cells and therefore have the potential of encountering viruses in the bloodstream, their fate is to contribute to the inflammation of the infected tissue. In fact, the priming of neutrophils in the circulation by an isolated stimulus is insufficient, and their complete activation to full capacity is a multistep process achieved after their transmigration through the endothelium following a chemotactic gradient (43). Secretion of IL-8/CXCL8, an important human neutrophil chemoattractant, was measured after mdDCs and PBMCs had been stimulated with ZIKV for 24 and 48 hours. LPS was used as a positive control of activation and boosted chemokine production (**Figure 5A**). A concentration of recombinant human IL-8 (rhIL-8) corresponding approximately to what had been detected in the mdDCs and PBMCs supernatant (1,666 pg/mL) was not enough to induce neutrophil migration in a transwell assay (**Figure 5B**). Neutrophils migrated with a rhIL-8 concentration that was 50 times higher (**Figure 5B**). Interestingly, when A549 cells, which are a more permissive cell line, were infected with ZIKV PE243 for 48 hours, they also did not promote neutrophil migration (**Figure 5B**).

### Human Neutrophils Reduce ZIKV Infection in Infected Cells by Cell-to-Cell Contact

**Figure 6:**
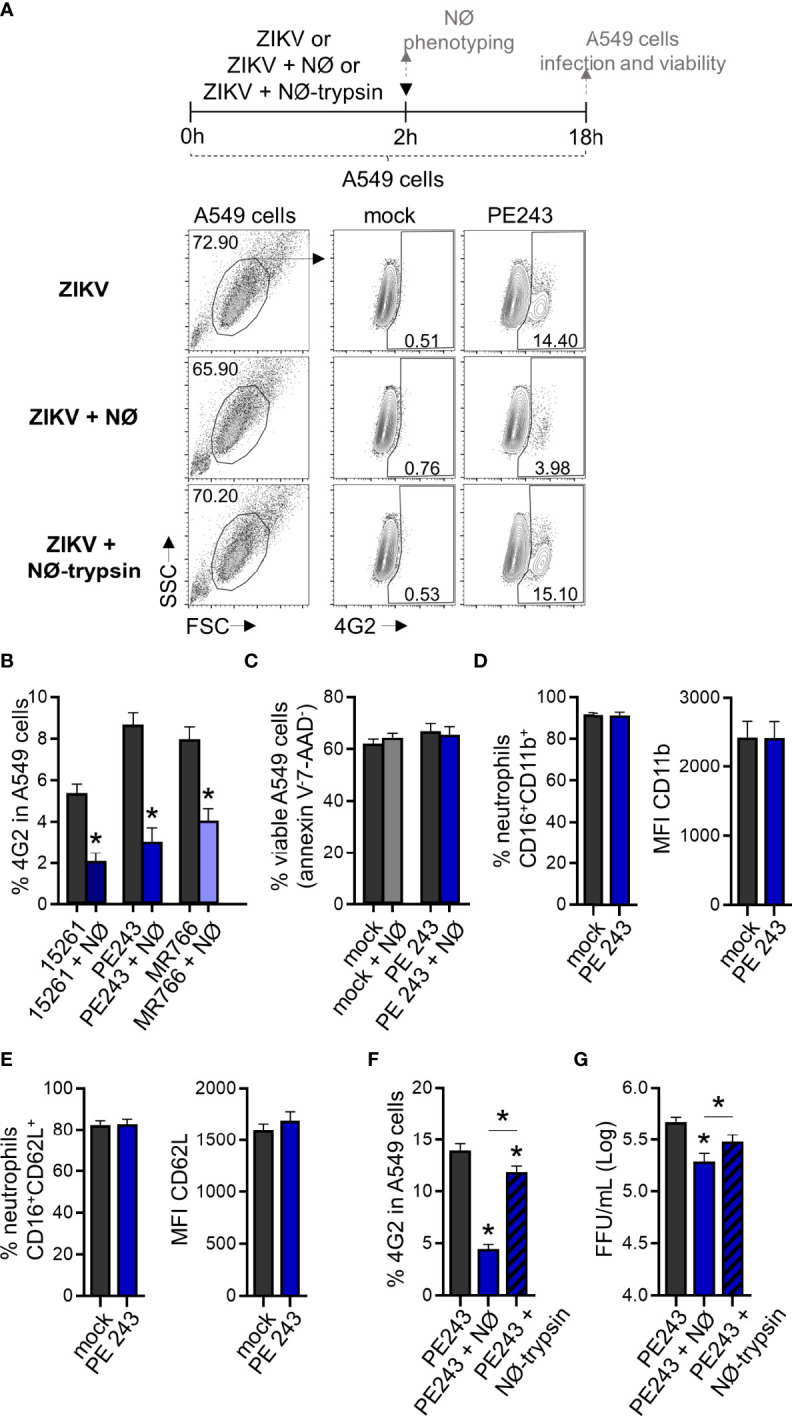
Human neutrophils reduce ZIKV infection in A549 cells through contact. **(A)** Flow cytometry plots depicting the frequency of 4G2^+^ A549 cells at 18 hours post-infection with ZIKV PE243 (1 MOI). Cells were infected for 2 hours in the absence of neutrophils (ZIKV), presence of neutrophils (ZIKV + NØ), or presence of neutrophils pretreated with trypsin (ZIKV + NØ-trypsin). ZIKV PE243 is shown as a representative of the ZIKV strains’ effects. Mock condition was used as a negative fluorescence control. **(B)** Frequency of 4G2^+^ A549 cells at 18 hours post-infection with ZIKV strains (1 MOI) in the absence or presence of neutrophils. **(C)** Frequency of viable A549 cells (annexin V^-^7-AAD^-^) after 18 hours of infection with ZIKV PE243 (1 MOI) in the absence or presence of neutrophils during the infection. **(D)** Frequency of neutrophils CD16^+^CD11b^+^ and the mean fluorescence intensity (MFI) of CD11b in that population after 2 hours of interaction with A459 cells stimulated with mock or PE243. **(E)** Same analysis as in **(D)** was applied to the CD62L molecule. **(F)** Frequency of 4G2^+^ A549 cells at 18 hours post-infection with ZIKV PE243 in the presence of trypsin pretreated neutrophils. **(G)** ZIKV PE243 loads on A549 cell culture supernatant at 18 hours post-infection in the presence of trypsin pretreated neutrophils. Bars indicate SEM. Three-four independent experiments are shown (n = 7–15). The asterisk (*) denotes the statistical difference between the absence and presence of neutrophils (B, F, G) or neutrophils pretreated with trypsin (F), and neutrophils pre-treated or not with trypsin. (▼) indicates the moment A549 cells were washed to remove the stimuli. NØ, neutrophils.

**Figure 7:**
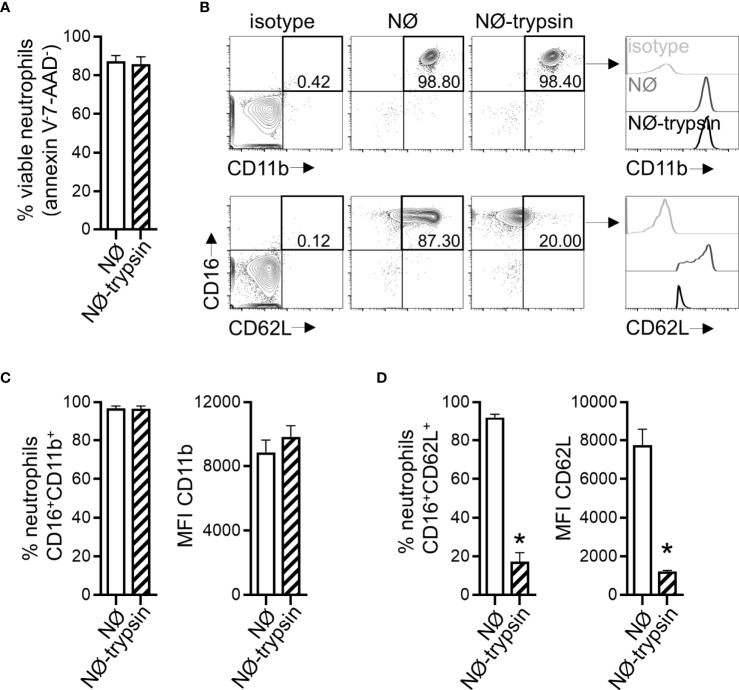
Trypsin treatment affects the expression of CD62L in neutrophils. **(A)** Frequency of viable neutrophils (annexin V^-^7AAD^-^) after treatment with trypsin. **(B)** Contour plots depicting the frequency of neutrophils CD16^+^CD11b^+^ and CD16^+^CD62L^+^ and the fluorescence intensity of CD11b and CD62L molecules inside these populations directly after isolation from blood (NØ) or in trypsin pretreated neutrophils (NØ-trypsin). Isotype control antibodies were used as a negative fluorescence control to set the gates. **(C)** Frequency of neutrophils CD16^+^CD11b^+^ and the mean fluorescence intensity (MFI) of CD11b in that population after treatment with trypsin. **(D)** Same analysis as in (C) was applied to the CD62L molecule. Bars indicate SEM. Two independent experiments are shown (n = 6–7). The asterisk (*) denotes the statistical difference between neutrophils treated with trypsin (D) and those not treated. NØ, neutrophils.

To mimic a situation where neutrophils reached an infected environment after migration, we co-cultured neutrophils with A549 cells. Neutrophils were added to A549 cells concomitantly to ZIKV infection. Both stimuli were maintained for 2 hours and then removed. The A549 cells infection frequency was evaluated 16 hours after removing the viral input through the detection of ZIKV E protein (4G2) (**Figure 6A**). A significant reduction (more than 50%) in A549 cell infection was observed when neutrophils were added during the infection period (**Figures 6A, B**). We did not detect a reduction in the frequency of A549 cells annexin V^-^7-AAD^-^ due to neutrophil presence (**Figure 6C**). Formerly, we hypothesized that a small number of virus particles might have been internalized by neutrophils, and a reduced fraction of the virus particles could be binding to the surface of the neutrophils. Nevertheless, such a small reduction in the number of free viral particles is not enough to explain the decrease in the infection rate observed in the co-culture. Besides, no decrease in A549 infection was detected when ZIKV was pre-incubated with neutrophils (**Figure 4**). Neutrophils co-cultured with A549 cells did not modulate CD11b and CD62L receptors (**Figures 6D, E**), indicating that reduced infection was not due to neutrophil activation. However, neutrophil treatment with trypsin before its addition to A549 cells restored their infection frequencies and replication (**Figures 6A, F, G**). Therefore, we hypothesized that neutrophils interacted with A459 cells through surface protein-membrane components and impaired ZIKV infection. The treatment with trypsin did not significantly affect the viability of neutrophils (annexin V^-^7-AAD^-^) (**Figure 7A**), nor the expression of CD11b (**Figures 7B, C**). However, it significantly reduced the expression of CD62L on neutrophils (**Figures 7B, D**), suggesting an impact on neutrophil surface proteins by trypsin. In an alternative co-culture setting, aimed to mimic the neutrophil role in an established ZIKV infection, A549 cells were infected for 24 hours with ZIKV strains; after this span of time, neutrophils were added to the culture for 16 hours. Even in a scenario where the infection was already established, a significant reduction in the frequency of ZIKV infection was observed after the addition of neutrophils (**Supplementary Figure 2**).

**Figure 8:**
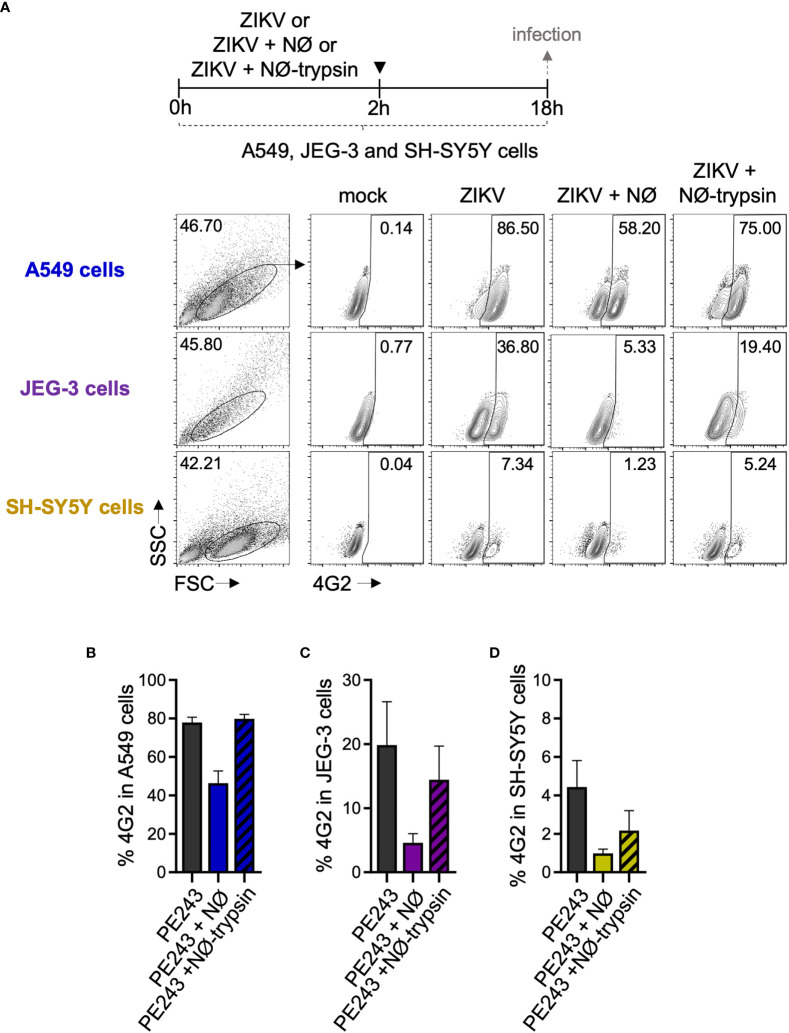
Human neutrophils reduce ZIKV infection in different cell lines. **(A)** Flow cytometry plots depicting the frequency of 4G2^+^ A549 cells, JEG-3, and SH-SY5Y cells 18 hours post-infection with ZIKV PE243 (10 MOI). Cells were infected for 2 hours in the absence of neutrophils (ZIKV-PE243), presence of neutrophils (ZIKV-PE243 + NØ), or presence of trypsin pretreated neutrophils (ZIKV-PE243 + NØ-trypsin). After the wash steps, cells were maintained for additional 16 hours. Mock condition was used as a negative fluorescence control. **(B)** Frequency of 4G2^+^ A549 cells 18 hours post-infection with ZIKV in the absence or presence of neutrophils. **(C)** Frequency of 4G2^+^ JEG-3 cells 18 hours post-infection with ZIKV in the absence or presence of neutrophils. **(D)** Frequency of 4G2^+^ SH-SY5Y cells 18 hours post-infection with ZIKV in the absence or presence of neutrophils. Bars indicate SEM. Two independent experiments are shown (n = 4–6). NØ, neutrophils.

Finally, in order to confirm that these findings are not restricted to A549 cells, we also performed co-cultures with other epithelial cell lineages, JEG-3 (placental cell) and SH-SY5Y (neuronal cell) (**Figure 8A**). **Figure 8B** shows that even when A549 were infected with a higher MOI (10 MOI), resulting in a massive infection, the addition of neutrophils to the culture reduced the frequency of ZIKV infected cells. Neutrophil treatment with trypsin before addition to A549 cells culture also restored the infection frequencies. The same trend was observed when JEG-3 (**Figure 8C**) and SH-SY5Y (**Figure 8D**) cells were evaluated. Although the frequency of ZIKV infected cells was lower than that found in A549 cells after 16 hours of infection, the addition of neutrophils almost eradicated the infection, and neutrophil trypsin treatment restored the infection levels (**Figures 8C, D**).

### Neutrophil Depletion Does Not Alter ZIKV Titers in the Draining Lymph Node

**Figure 9:**
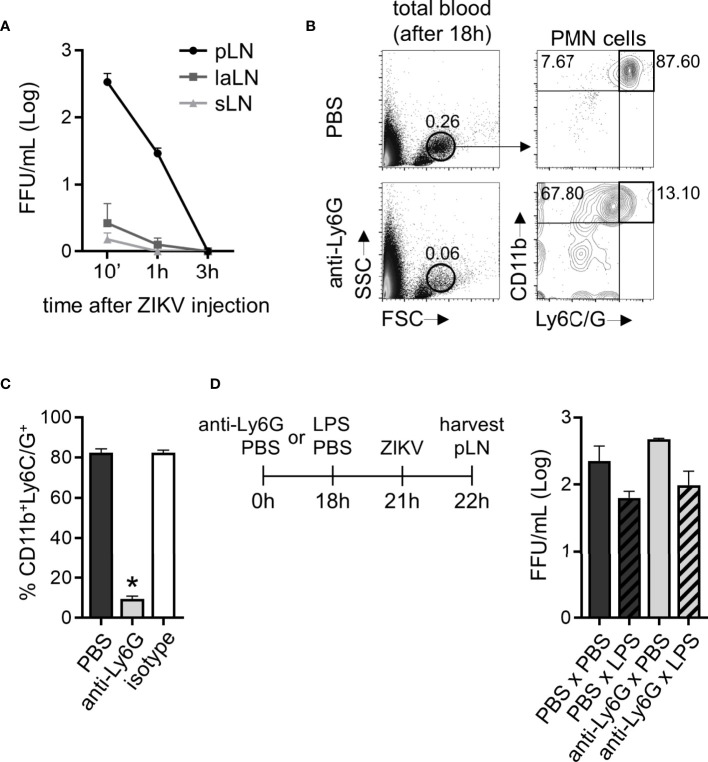
Mice neutrophil depletion does not restrict ZIKV relocation to draining lymph nodes. **(A)** ZIKV loads detected in the pool of both ipsi- and contralateral popliteal (pLN), sciatic (sLN), or lumbar aortic (laLN) lymph nodes per C57BL/6 mouse after 10 minutes, 1 and 3 hours of ZIKV PE243 (5x10^5^ FFU) injection to the footpad. **(B)** Flow cytometry plots depicting the frequency of PMN leukocytes CD11b^+^Ly6C/G^+^ in the total blood content of mice 18 hours post-treatment with PBS or anti-Ly6G antibody (400 µg). **(C)** Frequency of CD11b^+^Ly6C/G^+^ cells in the PMN population in mice treated with PBS, anti-Ly6G, or an isotype antibody for 18 hours. **(D)** After neutrophil depletion, mice received a footpad subcutaneous injection of PBS or LPS (1000 ng) and, 3 hours later, ZIKV PE243 (5x10^5^ FFU). One hour later, ipsi- and contralateral pLNs from each mouse were harvested and pooled to determine ZIKV loads. Bars indicate SEM. Three animals per group were used in each experiment. One of three independent experiments is shown. The asterisk (*) denotes the statistical difference between PBS and anti-Ly6G groups.

Following subcutaneous infection to the hind footpad, ZIKV PE243 was detected in the lymph nodes (popliteal (pLN), lumbar aortic (laLN), and sciatic lymph nodes (sLN)) of C57BL/6 immunocompetent mice only up to 3 hours after they had been infected (**Figure 9A**). ZIKV PE243 was not detected in the spleen, kidney, or blood of these animals in any of the assessed times. Therefore, in our subsequent studies, we used the pLN after 1 hour of ZIKV infection as a viral spread indicator site. C57BL/6 mice had significantly reduced CD11b^+^/Ly6C/G^+^ cells through pre-treatment with a monoclonal antibody targeting Ly6G (**Figures 9B, C**). Although a neutrophil-specific monoclonal antibody (anti-Ly6G clone 1A8) has been used for the depletion protocol, we cannot rule out the possibility of its impact on the monocyte population since depletion validation was carried through CD11b and Ly6C/G staining (**Figure 9C**). After 18 hours of neutrophil depletion treatment and 3 hours prior to ZIKV PE243 subcutaneous infection, mice were inoculated subcutaneously in the hind footpad with LPS to stimulate cell migration to the injection site (**Figure 9D**). The animals treated with the anti-Ly6G antibody (Ly6G x LPS) presented similar titers of ZIKV in pLN to animals that received no antibody treatment (PBS x LPS) (**Figure 9D**). Although, there seems to be a trend (not statistically significant) of higher viral titers in lymph nodes of neutrophil depleted mice (PBS x PBS vs. anti-Ly6G x PBS and PBS x LPS vs. anti-Ly6G x LPS). The slightly reduced titers in pLN seen in mice that received LPS compared to those that received PBS in the footpad were attributed to the inflammatory context. This result suggests that, in this model, the presence of neutrophils was not essential for containing the spread of the ZIKV to the draining lymph node.

## Discussion

Neutrophil-inflammatory responses triggered by viral infections are necessary for an effective antiviral immunity (48–51) but can also become dysregulated and result in tissue injury (52, 53). This concept could be extended to ZIKV pathology, in which neutrophils could be associated with the virus’ neurotropic nature, the ability to cause injury to the reproductive tract and be sexually transmitted, and enables the long-term viral persistence in some body fluids and tissues (3). In order to investigate these possibilities, we assessed the response of human neutrophils to Asian and African ZIKV strains under diverse experimental settings.

Neutrophils were suggested to be permissive to ZIKV as viral RNA was found in myeloperoxidase^+^ neutrophils present in the lymph nodes of cynomolgus monkeys 7 days post-infection (10) and in CD45^+^CD11b^+^ neutrophil-myeloid cells in the placenta of AIR mice (vertical transmission model in a *Rag1-*deficient mouse) (26). In contrast to those previous findings, our results show that, aside from some ZIKV RNA detection, human neutrophils do not appear to support any significant ZIKV replication and do not represent a noteworthy ZIKV reservoir. This is in line with previous reports that demonstrated that ZIKV preferentially targets CD14^+^CD16^+^ monocytes in the blood (16). Furthermore, we found AXL, a TAM family tyrosine kinase that has been described as a facilitator of ZIKV infection due to attenuation of type I IFN (54), was found to be absent from neutrophils’ surface. Nevertheless, ZIKV receptors and co-receptors are still not well characterized, and AXL is just one of the cell surface molecules that could help to mediate ZIKV infection.

Surprisingly, stimulation of neutrophils with ZIKV did not promote a strong cell activation or any classical neutrophil microbicidal mechanism, as noted by the absence of a robust CD11b and CD62L modulation, secretion of inflammatory cytokines and elastase, and the production of ROS and NETs. These results indicate that neutrophils presented a non-responsive phenotype in our experimental settings and therefore would not be the major source of inflammatory mediators during ZIKV infection. Other viruses, such as *Human immunodeficiency virus-1*, induced activation on neutrophils by modulating the expression of several Toll-like receptors, CD11b and CD62L, promoting the secretion of IL-6 and TNF-α, and altering ROS production (55). In accordance with our results, ZIKV and *Dengue virus* type 2 were previously shown to be unable to induce NETs in mice neutrophils (56). Interestingly, our data pointed out that ZIKV particles are not captured by NETs in the context of the DNA web induced by a secondary stimulus. Zanluqui et al.’s preprint manuscript (57) also addresses the role played by neutrophils during ZIKV infection. Corroborating our findings, the authors demonstrated the lack of ZIKV interference in human neutrophil viability and NETs release. Also, mice neutrophils, did not display a pro-inflammatory profile and ROS production in response to the virus (57). A viral escape from innate immune components could result in delayed immune responses favoring viral spread. We cannot rule out a possible suppression of neutrophil action by ZIKV, as previously shown for primary monocytes, mdDCs, and plasmacytoid DCs that have their maturation and activation impaired during ZIKV replication (14, 16, 17, 58, 59). Moreover, a cohort of rhesus monkeys produced minimal systemic cytokine response to ZIKV infection (9).

Several reports have indicated that neutrophils migrate to different tissues during ZIKV infection in IFN receptor-deficient mice, such as CNS, spleen, spinal cord, epididymis, and testis (19, 20, 24, 60, 61), and in humans and non-human primates’ mucosa, placenta, and fetus (11, 62, 63). Patients during the acute or recovery phases of the disease secrete increased levels of IL-8/CXCL8 (12, 13). *In vitro* assays with primary monocytes and THP-1 cells following ZIKV infection also showed increased amounts of IL-8 (16, 64). Moreover, myeloid cells from AG129 mice infected with ZIKV were responsible for the production of cytokines involved in leukocyte recruitment and viral dissemination to peripheral organs (23). Despite these reports, under our settings, ZIKV infection of mdDCs and PBMCs for 48 hours did not induce sufficient levels of IL-8 to promote neutrophil migration *in vitro*. Even in ZIKV infected A549 cells, which endure high levels of viral replication and can generate a more complex environment, neutrophil migration is not promoted. Frumence et al. (65) have shown low secretion levels of soluble IL-8 in ZIKV infected A549 cells, confirming that the *in vitro* assay we used has limitations in promoting neutrophil migration.

To assess the putative impact of neutrophil migration to infected tissues *in vitro*, we used a co-culture system with A549 cells. Our results show a reduction in the rate of ZIKV infection of A549 cells when neutrophils are present at the moment of the infection or in a pre-established infection. This infection impairment does not seem related to neutrophil activation but to the physical interaction between surface molecules in both cells. Co-culture assays using JEG-3 cells, a placental cell lineage, and SH-SY5Y, a neuronal cell lineage, corroborate such findings, showing that neutrophil action in infected cells was not lineage-dependent. It has been shown that cell-to-cell contact between neutrophils and A549 cells is conducive to a proliferative effect on these cells involving the release of elastase and COX-2 products by neutrophils (66). An increase in the production of IL-6 and IL-8 by A549 cells (67) and the induction of A549 cell death by apoptotic neutrophils induced by soluble Fas ligand (68) have also been reported. However, this was not the case in our model, where neutrophil presence did not affect A549 cell viability. The loss of CD62L in neutrophils after treatment with trypsin, a condition in which the frequency of ZIKV infection was restored in A549, JEG-3 and SH-SY5Y cells, might be related to the pathways involved in the neutrophil modulation of ZIKV infection in these cells. Herbert et al. (69) have reported that the β2- integrin ligand LFA-1 on neutrophils binds to the ICAM-1 receptor on epithelial cells and mediates, at least in part, epithelial damage, neutrophil degranulation, and reduction of the *Respiratory syncytial virus* (RSV) load. It remains to be determined what molecules and pathways might be involved during these cell-to-cell interactions that potentially contribute to the reduction of viral infection and proliferation; these could then be used in the future as targets against flavivirus infection. A secondary action of neutrophils at the site of infection is linked to the components of the mosquito’s saliva. During a *Semliki Forest virus* infection, the mosquito bite induced a neutrophil influx at the site of the bite, and these cells helped coordinate the entry of susceptible myeloid cells that are permissive to viral infection (70).

Finally, to better understand the role of neutrophils in ZIKV clearance at the inflammation site, we depleted neutrophils from C57BL/6 mice. In this scenario, we did not observe a direct action of neutrophils in preventing the spread of ZIKV to the lymph nodes during the first hour following ZIKV inoculation in a setting where an inflammatory environment had already been induced by LPS injection. Interestingly, other authors have reported that, apart from promoting an extensive recruitment of neutrophils to the inflammation site after certain virus infections (RSV, *Herpes simplex virus type 1*, and *Coxsackievirus B3*), neutrophils did not play an important role in viral replication and disease susceptibility, which was exerted instead by monocytes and macrophages (71–74).

Immunocompetent mice, such as those of the C57BL/6 strain, have been used for the Zika model (20, 75–77) but are reliant on the route of infection, virus strain, infection dose, and age, they could readily resolve ZIKV infections and might be a limited model for answering long-term questions. Intracranial ZIKV infection in C57BL/6 WT or Rag1^-/-^ mice (deficient in mature T and B cells) resulted in lethal encephalitis with infiltration of macrophages and NK cells (19). Neonatal immunocompetent mice challenged subcutaneously with ZIKV elicit CD8^+^ T cells recruitment to the CNS (20). Conversely, IFNAR^-/-^ mice (IFN type I and II receptor-deficient) infected by both routes have shown an accelerated spread of ZIKV to peripheral organs and to the CNS, where it elicits an inflammatory response characterized by neutrophil infiltration (19, 20). However, IFN pathway deficient models, despite being valuable tools for studying ZIKV pathology, could lead to changes in the natural pathogenic mechanisms, including the kinetics of neutrophil recruitment. Different studies have also reported enhanced recruitment of neutrophils to inflammation sites in IFNAR-deficient mice (20, 78–80). It would be of considerable interest to assess the production of neutrophil chemoattractants following ZIKV infection in the CNS of humans *post mortem* since ZIKV antagonizes human IFN-I (81).

In conclusion, our results indicate that human neutrophils are mildly activated by direct contact with ZIKV. However, the direct interaction between ZIKV and neutrophils does not contribute to viral replication or to the inflammatory disease associated with the viral infection. Conversely, human neutrophils are able to reduce ZIKV infection and replication on susceptible cell lines. However, the mechanistic role of neutrophils in this context is not yet clear. Finally, despite not being a target cell for ZIKV infection, our data suggest that, *in vitro*, neutrophils play a role in shaping ZIKV infections in other target cells.

## Data Availability Statement

All relevant data is contained within the article. The original contributions presented in the study are included in the article/**Supplementary Material**. Further inquiries can be directed to the corresponding authors.

## Ethics Statement

The studies involving human participants were reviewed and approved by Conselho Nacional de Ética em Pesquisa-CONEP (CAAE 60643816.6.0000.5248). The patients/participants provided their written informed consent to participate in this study. The animal study was reviewed and approved by Committee on the Ethics of Animal Experimentation from Fundação Oswaldo Cruz – CEUA/FIOCRUZ (license LW 03-19).

## Author Contributions

JA and PW conceptualized, designed, and performed the experiments and data analysis. BP and AM contributed to the interpretation and discussion of the data. CD contributed with her expertise in virology. All authors were involved in the writing of the manuscript.

## Funding

This research was funded by the Conselho Nacional de Desenvolvimento Científico e Tecnológico (CNPq-Universal - 444857/2014-1) and by Instituto Carlos Chagas/Fiocruz-PR (CNPq – PROEP-ICC 442356/2019-6). CS (307176/2018-5) is a CNPq fellow.

## Conflict of Interest

The authors declare that the research was conducted in the absence of any commercial or financial relationships that could be construed as a potential conflict of interest.

## Publisher’s Note

All claims expressed in this article are solely those of the authors and do not necessarily represent those of their affiliated organizations, or those of the publisher, the editors and the reviewers. Any product that may be evaluated in this article, or claim that may be made by its manufacturer, is not guaranteed or endorsed by the publisher.
