# Supplementary material for: Human Neutrophils Present Mild Activation by Zika Virus But Reduce the Infection of Susceptible Cells
Source: Front Immunol. 2022 Jun 7;13:784443. doi: 10.3389/fimmu.2022.784443 (PMC9210994; doi:10.3389/fimmu.2022.784443)
Supplement: Supplementary file 1 [file DataSheet_1.docx]

**Supplementary Material**

## Supplementary Figures


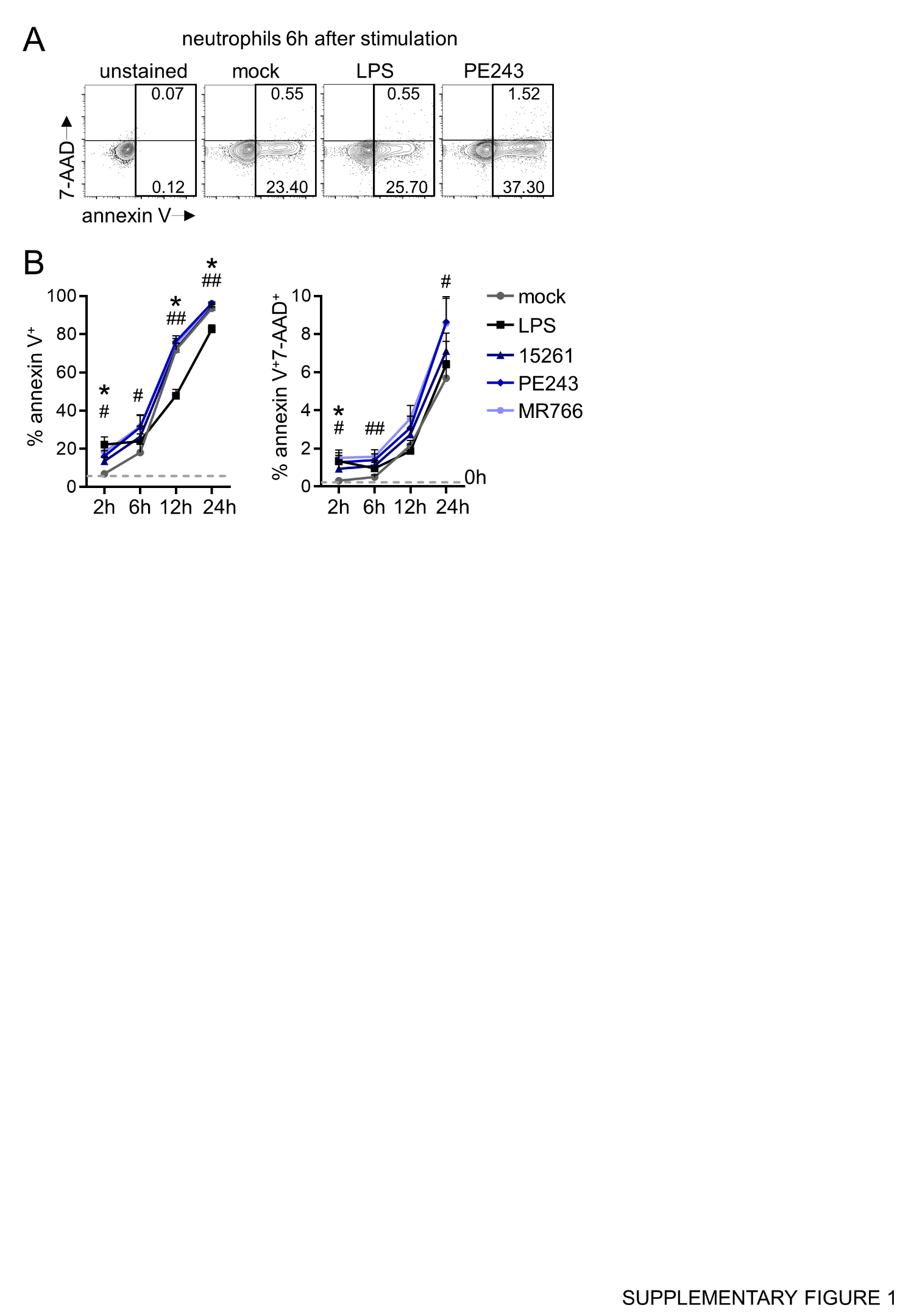


**Supplementary Figure 1.** ZIKV does not affect human neutrophil viability. **(A)** Contour plots depicting the frequency of annexin V^+^ and 7-AAD^+^ in the neutrophils gated population at 6 hours of stimulation with mock, LPS (100 ng/mL), or ZIKV PE243 (1 MOI), as a representative of the results. Mock-unstained condition was used as a negative fluorescence control to set the gates. **(B)** Frequency of annexin V^+^ and annexin V^+^7-AAD^+^ neutrophils at 2, 6, 12, and 24 hours of stimulation with mock, LPS, or ZIKV strains (1 MOI). The dashed line represents annexin V^+^ and annexinV^+^/7-AAD^+^ frequency right after neutrophil purification from blood (time 0). Bars indicate SEM. Three independent experiments are shown (n = 11). The asterisk (*) denotes the statistical difference between mock and LPS, and the number sign (#) is the difference between mock and all three ZIKV strains at that time point. Double number signs (##) denote the statistical difference between mock and ZIKV 15261 and MR766 (annexin V^+^ – 12 and 24 hours) and between mock and ZIKV PE243 and MR766 (annexin V^+^7-AAD^+^ – 6 hours).


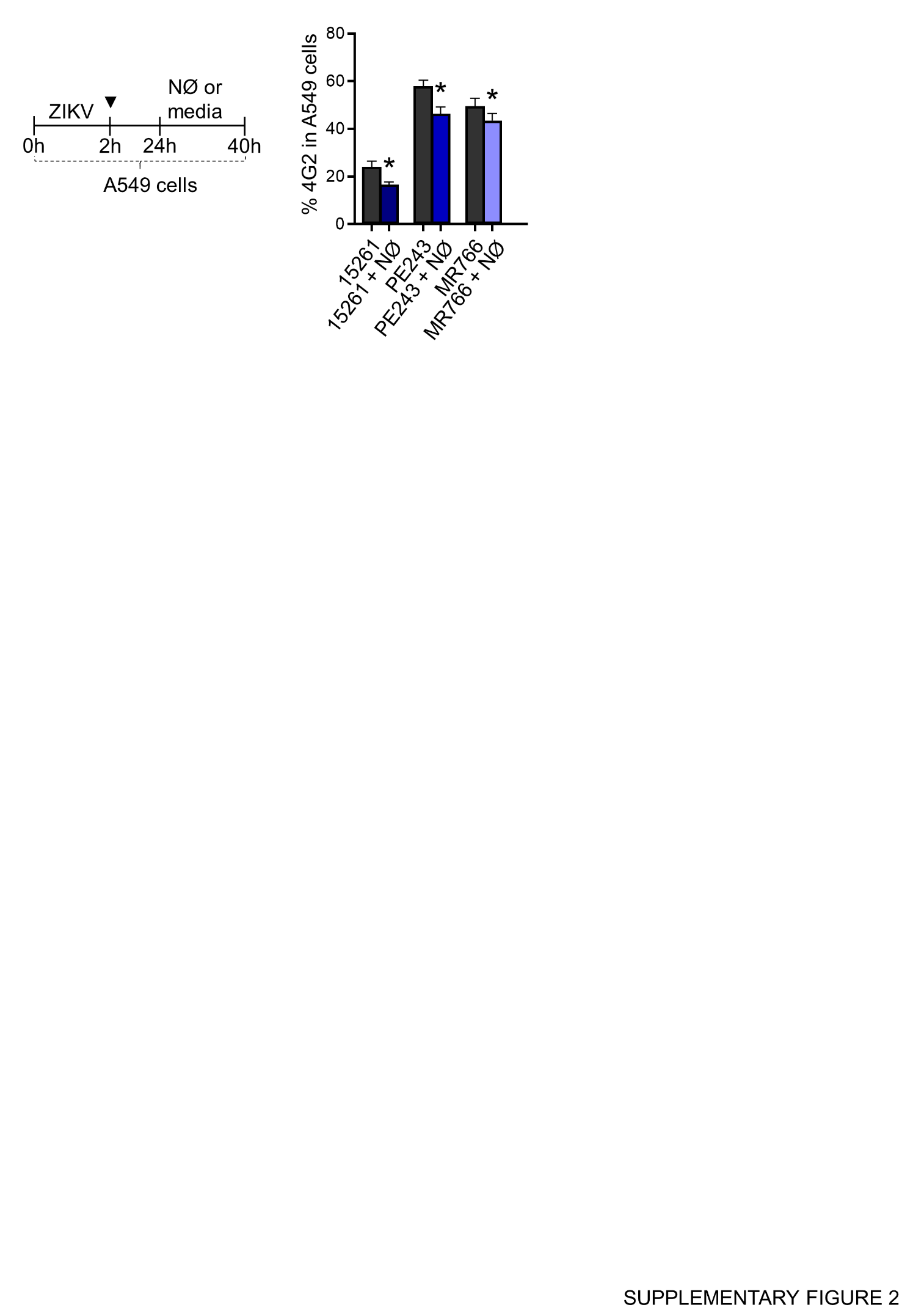


**Supplementary Figure 2.** Human neutrophils reduce ZIKV infection in A549 cells previously infected with ZIKV. Frequency of 4G2^+^ A549 cells at 40 hours post-infection with ZIKV strains (1 MOI) when neutrophils were added or not to the previously infected A459 cells at 24 hours post-infection and left in contact with the culture for an additional 16 hours. Bars indicate SEM. Three-four independent experiments are shown (n = 7-15). The asterisk (*) denotes the statistical difference between the conditions in which neutrophils were present or absent. NØ = neutrophils.
